# Point-of-Care Ultrasound in the Diagnosis of Pulmonary Embolism With Right Heart Strain: A Case Report of Dialysis Line-Related Complications

**DOI:** 10.7759/cureus.96273

**Published:** 2025-11-07

**Authors:** Oussama Medjahed, Avinash K Jha, Ahmed Ahmed, Ayman Shaat

**Affiliations:** 1 Critical Care, Royal Preston Hospital, Lancashire Teaching Hospitals NHS Foundation Trust (LTHTR), Preston, GBR; 2 Emergency Medicine, Royal Preston Hospital, Lancashire Teaching Hospitals NHS Foundation Trust (LTHTR), Preston, GBR

**Keywords:** dialysis access complications, haemodialysis (hd), hero graft, point-of-care ultrasound (pocus), pulmonary embolism (pe)

## Abstract

Pulmonary embolism (PE) represents a significant cause of preventable death among critically ill patients, with the immediate diagnosis impaired by the presence of hemodynamic compromise and limited access to advanced imaging. Point-of-care ultrasound (POCUS) is a rapid bedside tool for diagnosing right heart strain, which can aid in immediate clinical management. Here we report a case of a 57-year-old man with end-stage renal disease who was on hemodialysis through a Hemodialysis Reliable Outflow (HeRO) graft and presented with acute hypoxia and cardiovascular collapse after recent orthopedic surgery. Bedside POCUS showed a dilated, hypokinetic right ventricle and a mobile echogenic mass adjacent to the tricuspid valve, concerning for thrombus and device-related PE. Due to the subsequent hemodynamic instability, systemic thrombolysis was performed during cardiac arrest with return of spontaneous circulation. Follow-up CT pulmonary angiogram revealed extensive bilateral PE with right heart strain, and transesophageal echocardiography (TEE) showed the HeRO graft tip abutting the tricuspid valve with no evidence of thrombus attached to the tricuspid valve. Following a multidisciplinary discussion, the HeRO graft was removed, and therapeutic anticoagulation was initiated, resulting in complete resolution. This case highlights the life-saving role of POCUS in promptly screening and guiding the emergent management of PE when standard imaging is unavailable. It also demonstrates an uncommon but potentially life-threatening HeRO graft-related complication: intracardiac thrombus formation with PE, and its features emphasize the importance of heightened clinical suspicion and a multidisciplinary approach for such patients.

## Introduction

Pulmonary embolism (PE) is a condition with a significant, although reversible, cause for mortality in critically ill patients. The untreated patient population carries a risk of 30% mortality, while those treated have a mortality of around 8% [1]. In a post-mortem study, it was found that only a third of PE cases were diagnosed in patients in the intensive care unit (ICU) [1-3]. Early diagnosis is often challenging due to patient instability, causing delayed access to definitive imaging. Although point-of-care ultrasound (POCUS) has emerged as a valuable bedside screening tool, it has limitations for confirming PE. However, features such as right heart strain, a larger right ventricular dimension than the left ventricle, and McConnell's sign can be key indicators of acute PE and facilitate time-critical therapeutic decisions [4]. 

Patients on hemodialysis are at twice the risk of suffering from PE and a higher mortality risk compared to those without kidney disease, mainly due to the frequent use of central venous catheters and grafts [5]. The Hemodialysis Reliable Outflow (HeRO) graft, often used in cases of central venous occlusion, terminates in the right atrium. While providing durable access, it may serve as a nidus for intracardiac thrombus formation, leading to PE - a rare but potentially fatal complication [6]. In a study by Katzman et al., the incidence of HeRO graft-related right atrial embolus and possible PE was 2.6% [7]. Although rare, this complication can be fatal.

In this case, POCUS of the heart identified right heart strain and an intracardiac mass near the tip of the HeRO graft, leading to accelerated thrombolysis and helping with subsequent device management. 

## Case presentation

**Figure 1:**
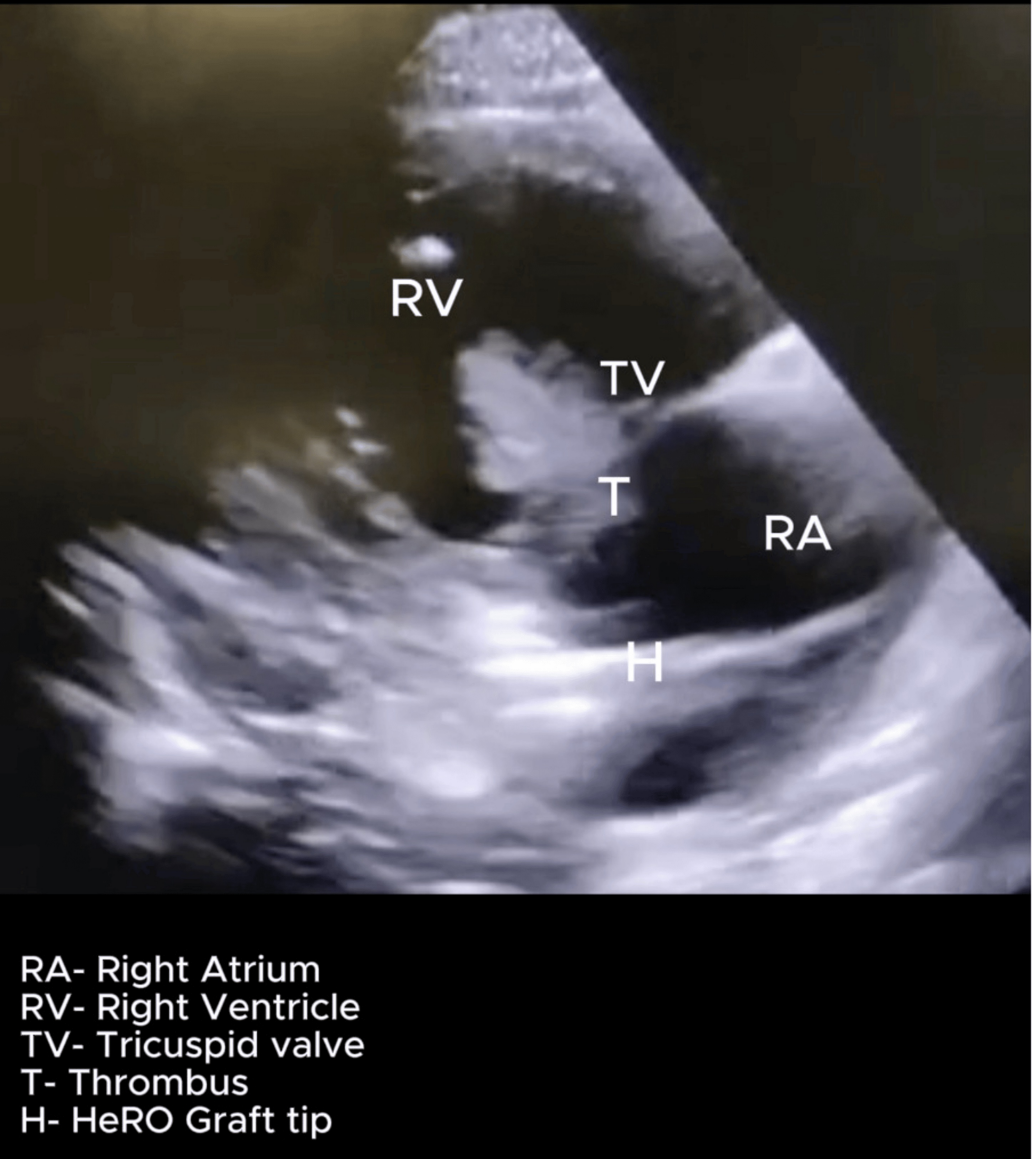
POCUS heart- right ventricle inflow view showing Hero graft tip and thrombus attached to tricuspid valve Probe: Phased array. View: Right ventricle inflow view. Depth: 19 cm POCUS, point-of-care ultrasound; HeRO, Hemodialysis Reliable Outflow

**Figure 2:**
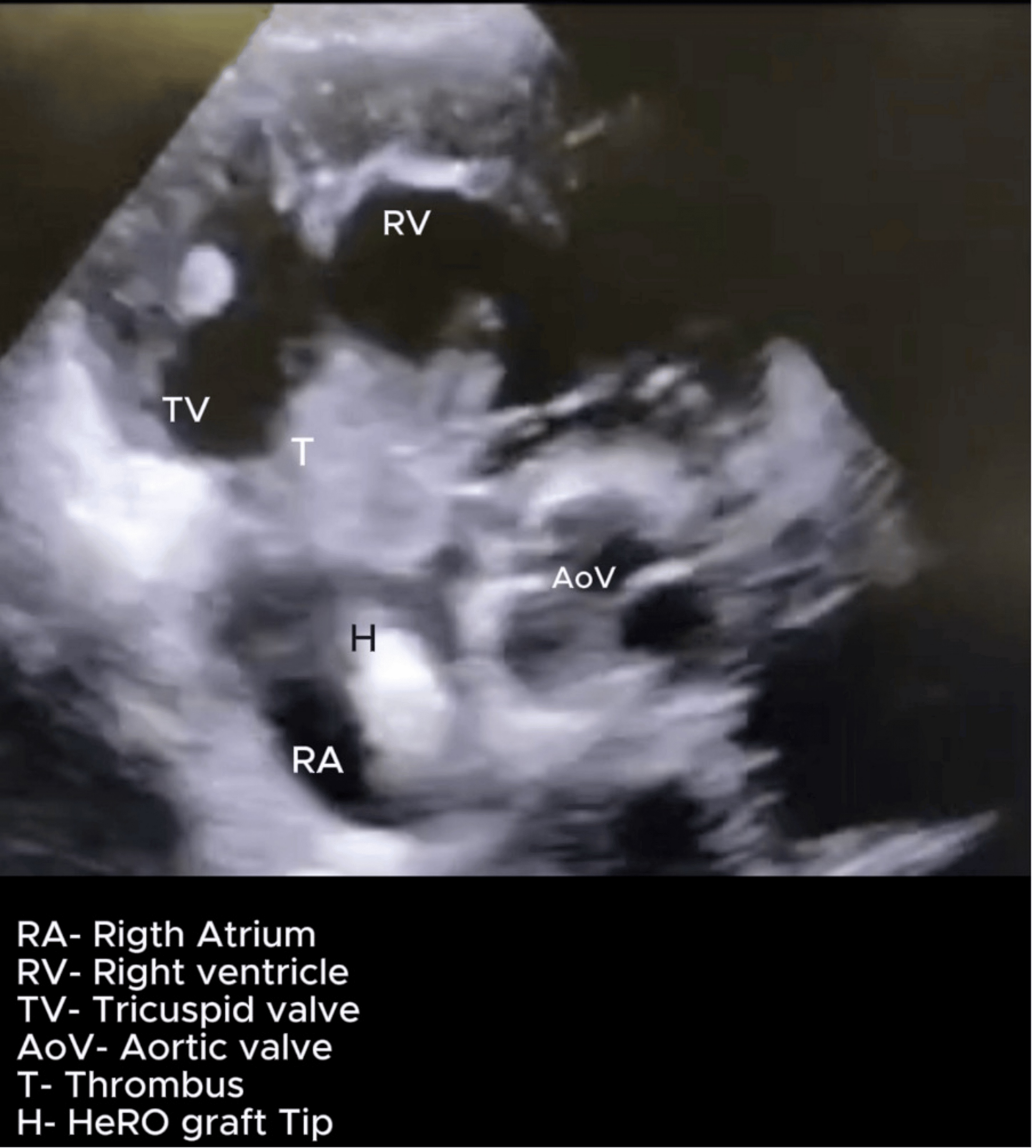
POCUS heart-parasternal short axis view at the aortic valve level showing HeRO graft tip and the thrombus attached to the tricuspid valve. Probe: Phased array. View: Parasternal short-axis view at the level of the aortic valve. Depth: 16 cm POCUS, point-of-care ultrasound; HeRO, Hemodialysis Reliable Outflow

**Figure 3:**
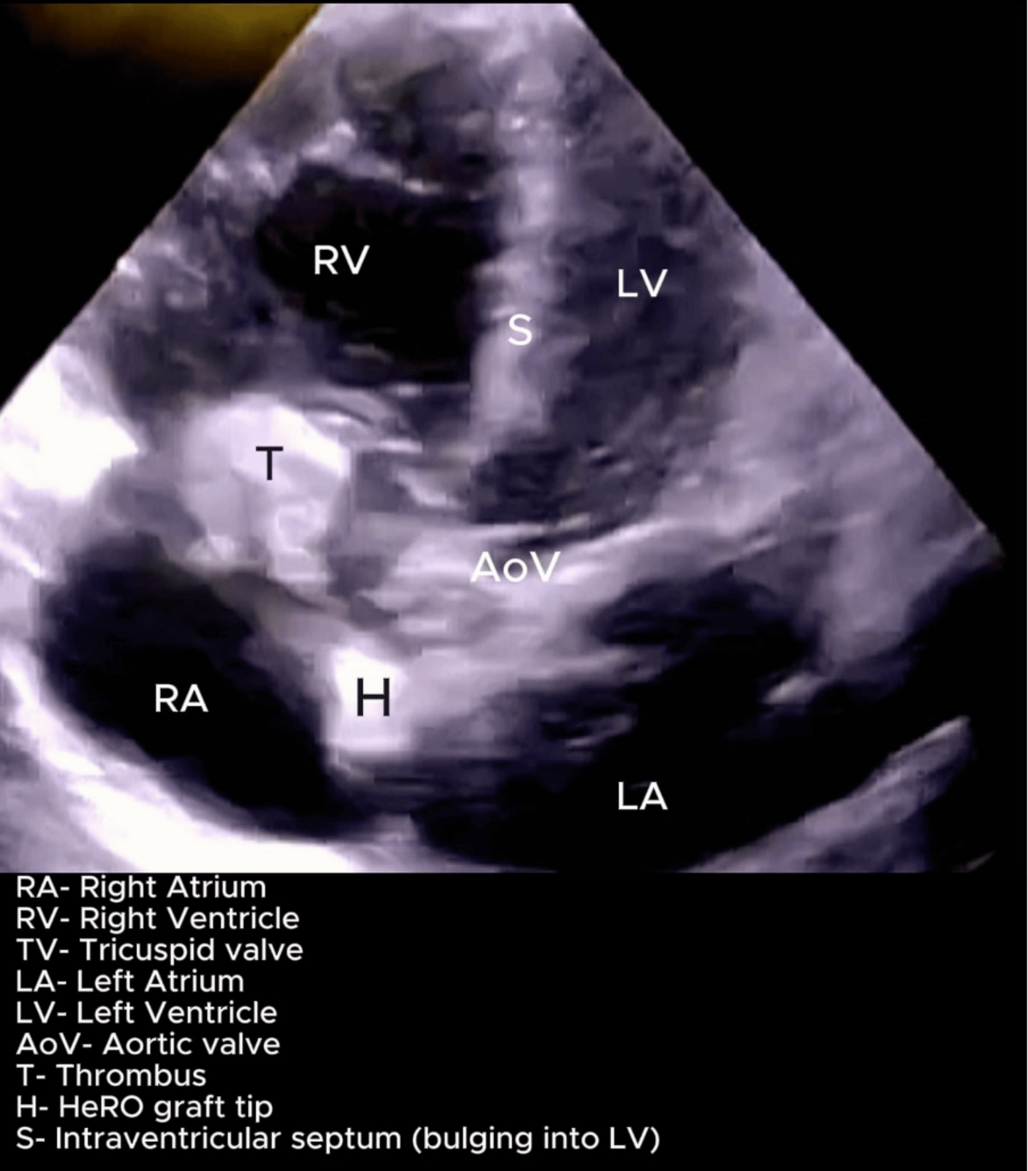
POCUS heart: Apical five-chamber view showing the HeRO graft tip and the thrombus attached to the tricuspid valve. Additionally, there are features of right ventricular strain with bulging of the interventricular septum into the left ventricle. Probe: Phased array probe. View: Apical five-chamber view. Depth: 18 cm. POCUS, point-of-care ultrasound; HeRO, Hemodialysis Reliable Outflow

**Figure 4:**
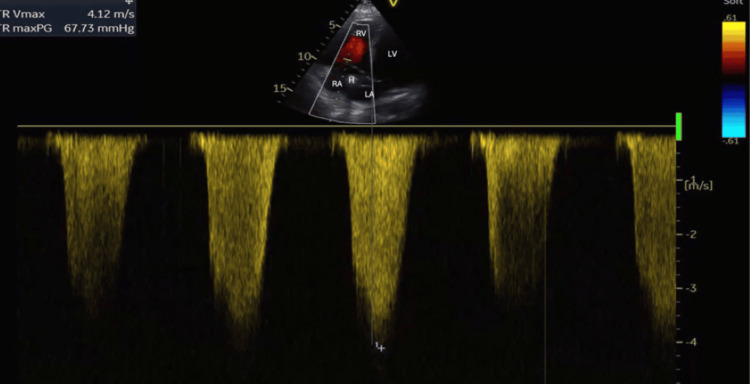
Estimation of pulmonary artery systolic pressure (PASP) assessed by point-of-care ultrasound of the heart. Probe: Phased array. View: Apical four-chamber view using continuous wave Doppler across the tricuspid valve. Depth: 17 cm. RA, right atrium; RV, right ventricle; LA, left atrium; LV, left ventricle; H, HeRO graft tip; TR Vmax, tricuspid regurgitation maximal velocity; TR maxPG, tricuspid regurgitation maximum pressure gradient

A 57-year-old man with end-stage renal disease (ESRD) of unknown cause, on thrice-weekly hemodialysis via a HeRO graft, was admitted after a mechanical fall. He was found to have bilateral quadriceps tendon rupture on imaging, attributed to chronic calcific tendinopathy. He underwent surgical repair on day 3 of admission to the hospital. Six hours post-procedure, he was commenced on enoxaparin 20 mg subcutaneously for deep venous thrombosis prophylaxis in view of ESRD, with anti-Xa monitoring targeting a peak concentration of 0.2 to 0.4 units/mL. His past medical history included hypertension, a prior PE, previously treated hepatitis C, and multiple failed vascular access attempts requiring HeRO graft placement. He was a non-smoker, lived independently, and had no recent signs of infection or thrombotic symptoms. On postoperative day 5 (day 8), he developed a sudden onset of confusion with tachypnoea with a respiratory rate of 28 cycles/minute and rising oxygen requirements. Examination revealed a blood pressure of 100/60 mmHg and hypoxia with increased work of breathing requiring supplemental oxygen by face mask. He was immediately transferred to the ICU due to these findings. A POCUS heart exam performed demonstrated a dilated and hypokinetic right ventricle and a positive McConnell sign. There was evidence of a dilated right atrium and a mobile, echo-dense mass near the tricuspid valve, suspicious for thrombus but not ruling out a vegetation. The HeRO graft tip was also visualized in the right atrium, suggesting a possibility of device-related thromboembolism, as shown in Figures 1-3. Additionally, key parameters included a tricuspid annular plane systolic excursion (TAPSE) of 10 mm, a right ventricle to left ventricle ratio of 1.4, and severe tricuspid regurgitation with an estimated pulmonary artery systolic pressure (PASP) of 80 mmHg (calculated using peak velocity of tricuspid regurgitation and estimated right atrial pressure), as seen in Figure 4. Videos 1-3 correspond to Figures 1-3, respectively.

**Video 1:** POCUS heart: right ventricular inflow view showing mobile thrombus attached to the tricuspid valve. The tip of the HeRO graft is also visualized in the right atrium. Probe: Phased array. View: Right ventricle inflow view. Depth: 19 cm POCUS, point-of-care ultrasound; HeRO, Hemodialysis Reliable Outflow

**Video 2:** POCUS heart: parasternal short-axis view (aortic valve level) showing mobile thrombus attached to the tricuspid valve. The HeRO graft can be visualized in the right atrium. Probe: phased array probe. View: Parasternal short-axis view at the level of the aortic valve. Depth: 16 cm POCUS, point-of-care ultrasound; HeRO, Hemodialysis Reliable Outflow

**Video 3:** POCUS heart: Apical five-chamber view showing mobile thrombus attached to the tricuspid valve along with HeRo graft in the right atrium. Additionally, right ventricular strain with bulging of the interventricular septum into the left ventricle is evident. Probe: Phased array probe. View: Apical five-chamber view. Depth: 18 cm POCUS, point-of-care ultrasound; HeRO, Hemodialysis Reliable Outflow

He was commenced on high-flow nasal cannula with a fractional inspired oxygen concentration of 50% and a flow rate of 40 L/minute. Paired blood cultures were obtained, and a loading dose of intravenous Vancomycin 2 grams and intravenous Gentamicin 210 mg was commenced as per hospital policy for infective endocarditis. In the following 10 minutes, the patient became profoundly hypotensive with a blood pressure of 70/40 mmHg and, unfortunately, suffered a cardiac arrest. Cardiopulmonary resuscitation (CPR) was initiated, and five minutes after its commencement, systemic thrombolysis was administered with an intravenous bolus of alteplase 10 mg, followed by 90 mg infused over two hours. Return of spontaneous circulation (ROSC) was achieved after 20 minutes. The patient was intubated and placed on mechanical ventilation during the cardiac arrest.

**Figure 5:**
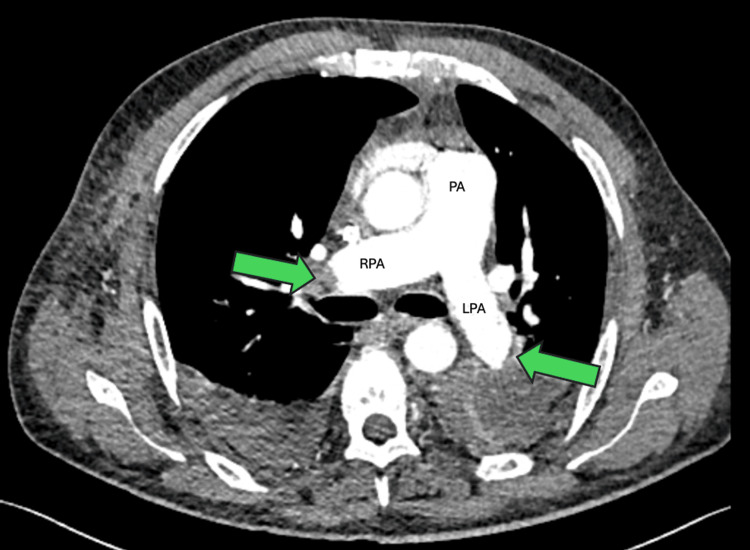
CT pulmonary angiogram (pulmonary arterial phase) showing thrombus occluding the right and left pulmonary arteries (green arrows pointing to the thrombus). PA, main pulmonary artery; RPA, right pulmonary artery; LPA, left pulmonary artery

After one hour of return of spontaneous circulation, the patient was stabilized hemodynamically on 0.05 microg/kg/minute of noradrenaline. He then underwent a CT pulmonary angiogram (CTPA) that revealed extensive bilateral PE (in the pulmonary arterial phase) with right heart strain (Figure 5). A transesophageal echocardiogram (TEE) was performed immediately after the CTPA by the cardiology team. This confirmed that the tip of the HeRO graft terminated at the tricuspid valve, contrary to the manufacturer’s guidance recommending tip placement in the upper atrium. The finding was best visualized in the mid-esophageal view focused on the tricuspid valve (Videos 4-5). Furthermore, there was no evidence of a mobile thrombus attached to the tricuspid valve on TEE.

**Video 4:** Transesophageal echocardiogram: Mid-esophageal view showing HeRO graft in the right atrium in close proximity to the tricuspid valve. The thrombus was not visualized post-thrombolysis. Probe: Transesophageal probe. View: Mid-esophageal view. Depth: 14 cm. Angle: 0 degrees. RA, right atrium; TV, tricuspid valve; RV, right ventricle; H, HeRO graft tip; IAS, interatrial septum; IVS, interventricular septum; LA, left atrium; MV, mitral valve; LV, left ventricle

**Video 5:** Transesophageal echocardiogram: mid-esophageal view focusing on the tricuspid valve showing the tip of the HeRO graft abutting the tricuspid valve. The thrombus was not visualized post-thrombolysis. Probe: Transesophageal probe. View: Mid-esophageal view. Depth: 14 cm. Angle: 122 degrees RA, right atrium; TV, tricuspid valve; RV, right ventricle; H, HeRO graft tip; HeRO, Hemodialysis Reliable Outflow

Intravenous vancomycin and gentamicin were continued with therapeutic drug monitoring. Given the echocardiographic findings and ongoing thromboembolic risk, a multidisciplinary team involving cardiology, nephrology, vascular surgery, and infectious diseases recommended removal of the HeRO graft based on the risk assessment. The rationale for the decision to remove the HeRO graft was the high risk of recurrence of thrombosis and subsequent PE. Hemodialysis was continued via a femoral hemodialysis catheter in the interim, inserted on postoperative day 5 (day 8) after ROSC. Anticoagulation was initiated with enoxaparin, along with anti-Xa level monitoring (targeting a peak concentration of 0.5 to 1 units/mL), considering recent surgery, after explaining the risk-benefit to the next of kin. No organisms were isolated in the three paired blood cultures. A repeat transthoracic echocardiogram (TTE) performed on postoperative day 6 (day 9) showed the absence of thrombus on the tricuspid valves, as seen in Videos 6-8. 

**Video 6:** A transthoracic echocardiogram performed after thrombolysis showed the tip of the HeRO graft abutting the tricuspid valve, with no thrombus attached to the valve. Probe: Phased array probe. View: Right ventricle inflow view. Depth: 20 cm. RA, right atrium; RV, right ventricle; TV, tricuspid valve; H, HeRO graft tip; HeRO, Hemodialysis Reliable Outflow

**Video 7:** A transthoracic echocardiogram performed after thrombolysis showed the tip of the HeRO graft in close proximity to the tricuspid valve, with no thrombus visualized on the valve. Probe: Phased array probe. View: Parasternal short-axis view at the aortic valve level. Depth: 16 cm RA, right atrium; RV, right ventricle; TV, tricuspid valve; AoV, aortic valve; H, HeRO graft tip; HeRO, Hemodialysis Reliable Outflow

**Video 8:** Transthoracic echocardiogram performed after thrombolysis showing the tip of the HeRO graft in close proximity to the tricuspid valve, with no clots visualized on the valve. Probe: Phased array probe. View: Apical four-chamber. Depth: 17 cm. RA, right atrium; RV, right ventricle; TV, tricuspid valve; LA, left atrium; LV, left ventricle; MV, mitral valve; H, HeRO graft tip; HeRO, Hemodialysis Reliable Outflow

Antibiotics were discontinued on postoperative day 7 (day 10) following hemodynamic stabilization, normalization of white cell count and C-reactive protein, and multidisciplinary discussion with the cardiology and microbiology teams. The patient was weaned off noradrenaline on postoperative day 7 (day 10) and extubated on postoperative day 9 (day 12). He was transferred to the ward under the renal team on postoperative day 10 (day 13) and subsequently underwent removal of the HeRO graft on postoperative day 20 (day 23). Therapeutic anticoagulation was continued after the procedure and later transitioned to warfarin, targeting an INR of 2-3 for long-term management. Figure 5 summarizes the key events of this case.

**Figure 6:**
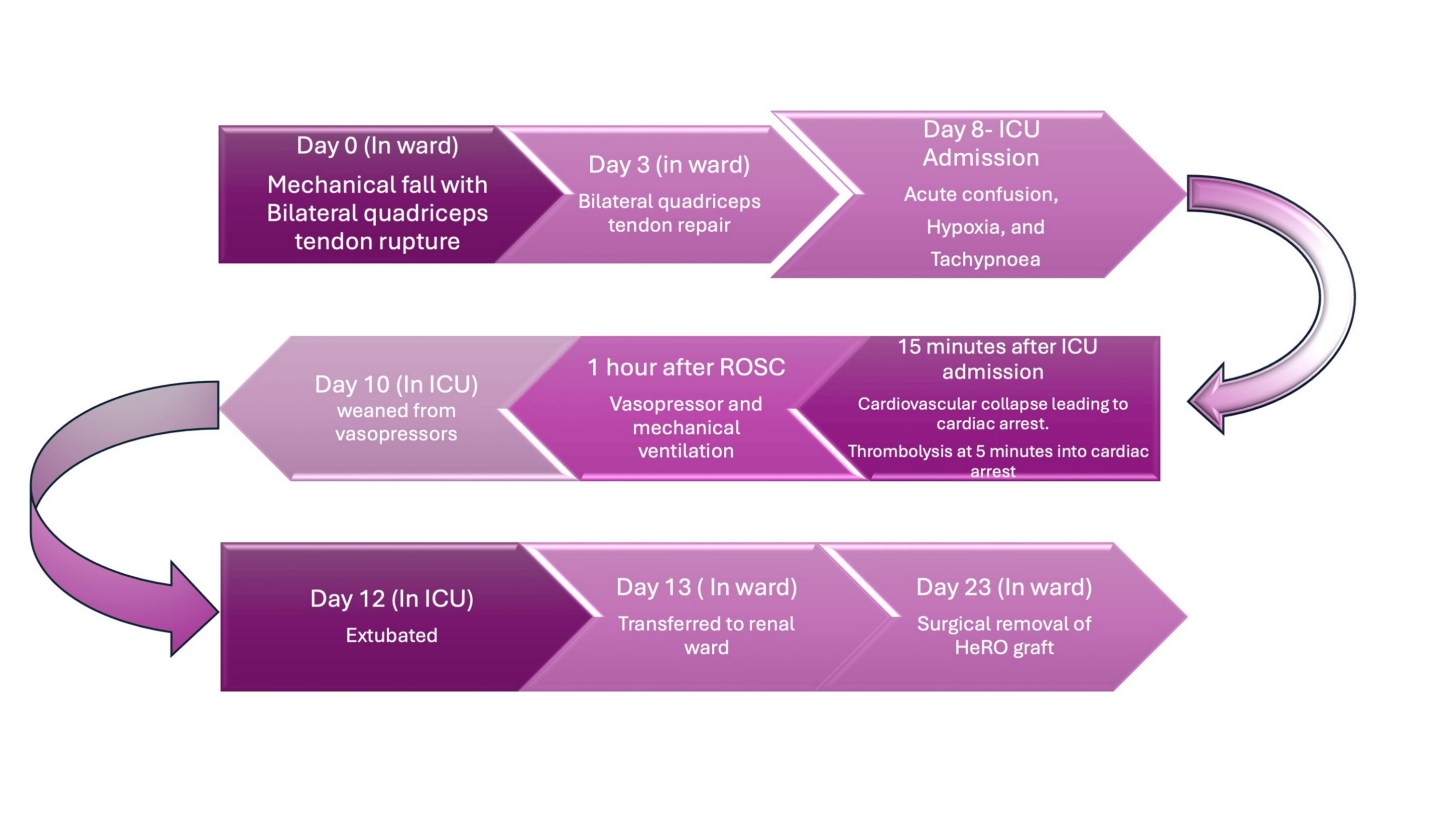
Summary of case events. The vasopressor used was norepinephrine. Image credit: Avinash K. Jha. ROSC, return of spontaneous circulation; ICU, intensive care unit

## Discussion

This case highlights the potentially life-saving importance of POCUS heart in evaluating and managing acutely unwell patients. In our case, bedside echocardiography identified right ventricular dysfunction and an intracardiac thrombus before confirmatory imaging (CTPA), which prompted emergency thrombolysis, urgent TEE, and subsequent removal of the HeRO graft. Although right heart strain alone is neither diagnostic nor sufficient for diagnosing PE, when it is accompanied by a visible thrombus and clinical decompensation, initiating emergency thrombolysis during cardiac arrest is justified. The literature on this subject is relatively limited, as most existing studies focus on POCUS in more stable patient groups.

The diagnostic yield we observed is consistent with the published literature for multi-organ POCUS in suspected PE. Melo et al. conducted a meta-analysis in critical care patients to evaluate the accuracy of multi-organ POCUS in PE. They reported a pooled sensitivity of 90% (95% confidence interval (CI) 0.85-0.94; *I*^2 ^= 0%) and a specificity of 69% (95% CI 0.42-0.87; *I*^2 ^= 95%) [8]. Similarly, another study evaluated 357 patients with multi-organ ultrasound examinations admitted to the emergency department, revealing a sensitivity of 90% and a specificity of 86.2% at diagnosing PE [9]. Our case demonstrates the role of POCUS in critically ill, hemodynamically unstable patients in the ICU, where cardiac POCUS has shown significant value not only for screening but also for immediate and long-term life-saving therapeutic decision-making.

This case also highlights a less-reported complication of the hemodialysis vascular access devices. The HeRO graft is designed to reduce infection rates compared to tunneled catheters, as demonstrated by Katzman et al., who reported a 69% lower infection rate. However, thromboembolic events were less well characterized [7]. Wu et al. demonstrated that thrombosed HeRO grafts have higher clot loads compared to conventional AV grafts and a higher PE risk during de-clotting [10]. At the same time, Sadjadi et al. reported a fatal PE after manipulation of the dialysis access [11]. In contrast to these cases, our patient developed spontaneous device-associated thromboembolism with no interventional trigger, with TEE demonstrating graft stent-related thrombus abutting the tricuspid valve. This reveals an under-appreciated risk of HeRO grafts and expands the list of its complications beyond infection and procedural thrombosis.

Device management and anticoagulation were also additional issues encountered in our case. While the 2019 KDOQI guidelines recommend individualized vascular access planning for complex patients with central venous occlusion, they offer minimal guidance on managing thrombogenic devices in the setting of PE [12]. Our case contributes to this body of evidence, illustrating that graft removal is a resource-intensive procedure that may be necessary to eliminate the ongoing risk of thromboembolism. In contrast to previously reported cases in which thrombectomy was the primary procedure, the joint decision to remove the HeRO graft represents a more definitive management choice.

Lastly, the difficulty in distinguishing thrombus from vegetation remains a significant limitation of POCUS of the heart. The mobile echodense material observed in bedside echocardiography could not be definitively characterized without the aid of TEE and a CT pulmonary angiogram. Additionally, the diagnostic accuracy of POCUS findings in isolation declines in the peri-arrest conditions, and the decision-making is based on clinical parameters and physiology. Our case highlights that while POCUS can prompt immediate, life-saving intervention, confirmatory imaging and microbiologic correlation are essential. The empirical use of both thrombolysis and antimicrobial therapy in our patient reflects a pragmatic approach to prioritizing survival when in doubt - a principle that aligns with and extends current guidelines.

## Conclusions

Our case underscores the vital role of POCUS of the heart in raising suspicion for potentially life-threatening PE in critically unstable patients when access to definitive imaging is delayed. However, CTPA and TEE were needed to confirm the diagnosis and make a timely decision regarding the subsequent management of the HeRO graft device.

Although HeRO grafts are typically utilized to provide infection-free vascular access in ESRD with central venous occlusion, our case demonstrates that when the graft’s outflow tip resides at or abuts the tricuspid apparatus, a high index of suspicion for device-related thrombosis is warranted, given the potential for embolic phenomena. A multimodal approach, complementing rapidly available POCUS findings with confirmatory imaging and early device consideration, remains vital to achieving optimal outcomes in such complex cases.
